# Evaluation of comprehensive care for older adults in primary care services

**DOI:** 10.11606/s1518-8787.2020054001370

**Published:** 2020-01-14

**Authors:** Nádia Placideli, Elen Rose Lodeiro Castanheira, Adriano Dias, Pedro Alcântara da Silva, Josiane Lozigia Fernandes Carrapato, Patricia Rodrigues Sanine, Dinair Ferreira Machado, Carolina Siqueira Mendonça, Thais Fernanda Tortorelli Zarili, Luceime Olivia Nunes, José Fernando Casquel Monti, Zulmira Maria de Araújo Hartz, Maria Ines Battistella Nemes

**Affiliations:** I Universidade Estadual Paulista. Faculdade de Medicina de Botucatu. Departamento de Enfermagem. Botucatu, SP, Brasil; II Universidade Estadual Paulista. Faculdade de Medicina de Botucatu. Programa de Pós-Graduação em Saúde Coletiva. Departamento de Saúde Pública. Botucatu, SP, Brasil; III Universidade de Lisboa. Instituto de Ciências Sociais. Lisboa. Portugal; IV Instituto Toledo de Ensino. Bauru, SP, Brasil; V Universidade Federal de São Carlos. Faculdade de Medicina. São Carlos, SP, Brasil; VI Universidade Nova de Lisboa. Instituto de Higiene e Medicina Tropical. Lisboa. Portugal; VII Universidade de São Paulo. Faculdade de Medicina. Departamento de Medicina Preventiva. São Paulo, SP, Brasil

**Keywords:** Health Services for Older Adults, Comprehensive Health Care, Primary Health Care, Health Services Research, Brazilian Unified Health System

## Abstract

**OBJECTIVE:**

To evaluate the performance of comprehensive care for older adults in primary care services in the Brazilian Unified Health System in the state of São Paulo, Brazil.

**METHODS:**

A total of 157 primary care services from five health regions in midwestern São Paulo responded, from October to December 2014, the pre-validated 2014 questionnaire for primary care services assessment and monitoring. We selected 155 questions, based on national policies and guidelines on this theme. The responses indicate the service performance in older adults’ care, clustered into three areas of analysis: health care for active and healthy aging (45 indicators, d1), chronic noncommunicable diseases care (89 indicators, d2), and support network in aging care (21 indicators, d3). Performance was measured by the sum of positive (value 1) or negative (value 0) responses for each indicator. Services were clustered according to k-means of the performance scores of each domain. After weighting the domains (Z tests), we estimated the associations between the scores of each domain and independent management variables (typology, planning and evaluation of services), with simple and multiple linear regression.

**RESULTS:**

Chronic noncommunicable diseases care (d2) showed, for all clusters, better average performance (55.7) than domains d1 (35.4) and d3 (39.2). Service performance in the general area of planning and evaluation associates with the performance of older adults’ care.

**CONCLUSIONS:**

The evaluated services had incipient implementation of comprehensive care for older adults. The evaluation framework can contribute to processes to improve the quality of primary health care.

## INTRODUCTION

Primary health care (PHC) is pointed out as a priority to assist and monitor older adults’ health status, besides acting in the prevention of health problems and in the health promotion to a healthy aging. Accordingly, the World Health Organization (WHO) proposed in 2004 that primary care services should adapt to serve older adults properly^1^. In Brazil, in view of the population aging, the Ministry of Health published in 2007 the *Caderno de Atenção Básica* (Primary Care Guide) no. 19, guiding primary care teams for a greater resolution of the older adults’ demands^2^.

The expansion of the PHC public network and the need to strengthen management structures and to redefine roles in the three spheres of government have driven the institutionalization of agreement and evaluation mechanisms to assist the decision–making process and to improve the public health system^3 , 4^. Especially since the last decade, evaluation and monitoring strategies are essential tools to measure the effectiveness of health systems at all levels of care^4^.

In this context, evaluating services for the older adults’ health care, especially in PHC, is increasingly important to know the advances in implementing the recommendations of the Primary Care Guide no. 19^2^. They cover promotion and prevention actions to achieve active and healthy aging, management of chronic noncommunicable diseases (CNCD) and networking.

National and international studies conducted to evaluate the older adults’ care in PHC indicate the need to expand preventive practices and health promotion, infrequent and low diverse in actions, in addition to the importance to build networks of comprehensive care to older adults’ health, ordained by PHC services^5^. The QualiAB 2014 instrument (Questionnaire for Primary Care Services Assessment and Monitoring) proposes, among its indicators for the comprehensive evaluation of primary care services, those directed to older adults’ care^11^.

Despite the political and technological propositions available in the country for PHC performance in this segment, literature on the actions effectively implemented evaluating their organization and supply is still scarce^5^, as well as on the challenges generated by population aging for these services^5^. Most studies evaluate the actions developed in PHC according to the older adults’ perception of the care they received, and those who take the organization of actions in a comprehensive way and directed to the work carried out by the teams as an evaluative focus are rare.

This study aims to evaluate the performance of comprehensive care for older adults in primary health care services according to their managers and professionals, as well as to analyze the relationship between performance and indicators of health planning and evaluation.

## METHODS

Cross-sectional evaluative research based on the analysis of the results of QualiAB application in 2014. The evaluation occurred in PHC services located in five health regions in midwestern São Paulo: Bauru, Jaú, Lins, Polo Cuesta and Vale do Jurumirim, totaling 68 municipalities and 303 services, according to CNES^12^ (eliminating duplicate and non–corresponding registrations to PHC services).

This is a convenience sample, consisting of five health regions chosen for a census application of QualiAB 2014. The regions are in areas close to the higher education institution responsible for the research, with which previous partnerships were made in projects to support the management and evaluation of PHC services using the same instrument^13^. A total of 157 services distributed in 41 municipalities adhered to the project, representing 63% of the existing in the period^12^. Adhesion was voluntary since no financial incentive or prize was offered in the process.

Teams of each health service answered QualiAB 2014 online, after the municipal manager and the managers or responsible for each service adhered. This application integrated the instrument upgrade and revalidation process, originally validated in 2007^13^.

This QualiAB version consists of 126 multiple–choice questions, which generate composite indicators for the overall evaluation of PHC services, to cover the diverse set of health actions attributed to primary care, as provided for the National Primary Care Policy (PNAB— *Política Nacional de Atenção Básica* )^16^. It is a questionnaire developed to evaluate and monitor services regarding the structure and organization of the work process by indicators of care and management, regardless of the type of unit, that is, organized both according to the Family Health Strategy and by another type of arrangement between the various existing compositions^13^.

To this study, we chose and categorized the variables of QualiAB 2014 related to older adults’ health and aging care, thus defining 155 performance indicators. First, we identified and analyzed documents on this evaluation object, to enable its delimitation and build a logical-theoretical model that will guide the selection of indicators and their organization in domains. Although a program specifically designed for the older adults’ health is not instituted, as it is with other segments, guidelines and technical standards are available, which allow delimiting the actions that should be developed at this level of care^17^.

We used the following documents: the Brazilian Health Policy for Older Adults (PNSPI— *Política Nacional de Saúde da Pessoa Idosa* )^18^, and the guidelines of the Ministry of Health published in the Primary Care Guide no. 19^2^, in the Strategic Action Plan to Tackle Noncommunicable Diseases (NCD) in Brazil 2011–2022^19^, and in the technical manual *Guidelines for the care of people with chronic diseases in Health Care Nets and in priority lines of care* ^20^.

The QualiAB 2014 selected indicators were gathered according to three large sets of attributes considered essential to comprehensive care for the older adults’ health and aging in three domains: health care for active and healthy aging (d1, composed of 45 indicators), chronic noncommunicable diseases (CNCD) care (d2, composed of 89 indicators), and structure and support network in the aging care (d3, composed of 21 indicators).

Statistical analysis was conducted from different strategies. To each domain, a final score equivalent to the sum of dichotomous responses was assigned to the indicators that compose it. Value 1 refers to achievement and value 0 to non-achievement. We also defined three clusters (performance groups) using k-means related to the scores, to achieve maximum heterogeneity between the different groups and maximum internal homogeneity in each group^21^. The three groups were organized by decreasing gradation of k-means obtained by the services, naming group 1 (G1) the services with the highest k-means, and group 3 (G3) those with the lowest k-means.

The second stage tested the association of each group composition with the responses to 30 QualiAB 2014 indicators that characterize the activities related to the service planning and general evaluation, shown in Chart 1 . The associations between the selected indicators and each of the three domains were estimated using chi-square tests followed by Z tests. As each domain consisted of different amounts of indicators (with maximum scores of 45, 89 and 21, respectively), the scores were standardized to keep them within the same magnitude scale.

**Chart 1:** Independent variables related to the planning and evaluation dimensions used in simple and multiple linear regression models, based on the questionnaire QualiAB 2014.

Dimension	Thematic cores	Independent variables
Planning	Type of service	- Family Health Unit (FHU) - Traditional Basic Health Unit (BHU) - BHU with Family Health Strategy (FHS) - BHU with Community Health Agents Program (CHAP) - BHU or FHU integrated to emergency care - Health outpost (PAS)
	Geographic location	- Rural - Central urban - Peripheral urban
	Planning of the service coverage area	- Area defined by participatory planning, considering local reality and ease of access - Area defined by the service team - No definition of the coverage area
	Matrix support	- Family health support center (FHSC) - Multiprofessional team - It is not carried out by any instance
Evaluation	Use of epidemiological data for planning	- By municipal management and unit, for planning - By municipal management, but inappropriate by the unit - Not used
	Participation in evaluations in the last three years	- Participated - Did not participate
	Developments based on the evaluations performed	- Annual teamwork plan - Activity planning and reprogramming with participation of the multiprofessional team - Report on the problems identified for the municipal secretariat - The service lacked access to the results - No evaluation was carried out
	Changes induced by evaluations	- Assistance organization - Service management - Assistance management and organization - No changes were reported - Did not participate in any previous evaluation

With the standardized scores, simple and multiple linear regression models were adjusted. As independent variables we had the service selected characteristics (Chart 1), and as response variables, the scores of each domain. The variables that obtained r^2^ values above 50% and significance levels of the model lower than 0.25 with normal distribution of residues in simple adjustments were carried to multiple adjustment, in which those with r^2^ above 80%, p < 0.05 values, and normal residues distribution were maintained. All steps of statistical analysis were developed with SPSS software version 20.0.

This study was approved by the Research Ethics Committee of the Faculdade de Medicina de Botucatu, Universidade Estadual de São Paulo by opinion no. 855,404.

## RESULTS

Among the 157 PHC services evaluated, according to self-classification, Family Health Units (FHU; 42%) prevailed, followed by traditional Basic Health Units (BHU; 36.9%). The Family Health Strategy (FHS) was present in 8.2% of the BHU, and the Community Health Agent Program (CHAP), in 8.9%; 1.2% were traditional BHU or FHU integrated to emergency care, and 2.5% of the services chose to be classified with other modalities.

All 45 QualiAB 2014 indicators elected to domain 1 (d1) cover actions of promotion, prevention and assistance related to older adults; aging care in the prevention of health problems and in health promotion; strategies and actions in situations of violence against older adults; and attention to older adults’ caregivers. Domain 2 (d2) clustered 89 indicators corresponding to routine actions for people with CNCD and strategies to approach non-adherence to treatment; programmatic care, routine examinations and medications available to people with arterial hypertension and to people with type 2 diabetes mellitus; and actions for bedridden people care. In domain 3 (d3), we clustered 21 indicators related to infrastructure, supplies and professional qualifications that enable prevention and health promotion for an active and healthy aging, in addition to the network of services for older adults’ health care in collaborative work with primary care. The services distribution in the different groups (G1, G2 and G3) and the average performance according to each analysis domain are shown in Table 1 .

**Table 1:** Performance of primary health and aging care services for older adults, clustered according to k-means, per analysis domain, 2014 (n = 157).

Domains	Groups	N	Mean	SD	Minimum	Maximum
Health care for active and healthy aging (d1)	1	20	68,4	9,0	47,5	83,1
	2	106	35,4	13,9	5,9	71,2
	3	31	12,0	5,3	1,9	23,7
Chronic noncommunicable diseases care (d2)	1	19	73,6	3,8	66,0	80,0
	2	105	55,7	8,0	37,0	71,0
	3	31	35,4	7,5	20,0	49,0
Structure and support network in aging care (d3)	1	20	51,0	11,1	33,9	67,8
	2	106	39,2	10,1	12,7	59,3
	3	31	30,2	8,8	12,7	50,8

SD: standard deviation

In all three areas most services are concentrated in group 2 of performance, although with variations between the means achieved. The highest values were obtained in the domain of CNCD care (d2), in which the highest means are also visible.

In domain 1, it is noteworthy the services presented the lowest means among the groups with worst performances. In domain 3, group 1 obtained its lowest mean (51.0) among all domains. Notably, most services perform less than half of the set of indicators evaluated, composing groups 2 (39.2; d2) and 3 (30.2; d3). The slightest difference between the means of the groups in this domain suggests the services show less differentiation in relation to the indicators.

Tables 2 and 3 show the frequencies of indicators by domain and by performance group (G1, G2 and G3), allowing to deepen the understanding of central tendency measures. The differences between groups are significant in 72.9% (113) out of the 155 indicators, shown in Table 2 . Services belonging to G1 have high percentages of achievement of most of the actions evaluated; however, they too lacks totality for a comprehensive care for older adults’ health and aging.

**Table 2:** Percentage distribution (%) of performance indicator frequencies in older adults’ health and aging care in primary care services, according to domain (d1, d2 and d3) and performance groups (G1, G2 and G3), with significant p-values, 2014 (n = 157).

Domain	Indicators	G1	G2	G3	p
d1	Cognitive evaluation	90.0_b_	38.7_c_	9.7_a_	0.001
	Depressive conditions evaluation	90.0_b_	43.4_c_	3.2_a_	0.001
	Functional evaluation	85.0_b_	19.8_c_	0.0_a_	0.001
	Fall prevention guidelines	95.0_b_	60.4_c_	0.0_a_	0.001
	Guidelines on food and nutrition	100.0_b_	72.6_c_	6.5_a_	0.001
	Guidelines on urinary incontinence	95.0_b_	48.1_c_	0.0_a_	0.001
	Guidelines on the importance of physical activity	95.0_b_	42.5_c_	0.0_a_	0.001
	Actions on sexuality and sexually transmitted diseases (STD)	90.0_b_	32.1_c_	0.0_a_	0.001
	Guidelines on menopause and andropause	95.0_b_	44.3_c_	6.5_a_	0.001
	Guidelines on oral health	95.0_b_	68.9_c_	16.1_a_	0.001
	Guidelines on older adults’ rights	70.0_b_	29.2_c_	3.2_a_	0.001
	Guidelines on violence against older adults	85.0_b_	32.1_c_	0.0_a_	0.001
	Investigation of alcohol and other drugs abuse	75.0_b_	23.6_c_	0.0_a_	0.001
	Home care	100.0_b_	59.4_a_	29.0_a_	0.001
	Support for older adults in long-term care facilities	50.0_b_	15.1_b_	9.7_a_	0.001
	Dental care for older adults	75.0_b_	52.8_a_	25.8_a_	0.002
	Health education in the community in the promotion of healthy aging	55.0_b_	21.7_c_	3.2_a_	0.001
	Health education in the service in the promotion of healthy aging	80.0_b_	22.6_c_	0.0_a_	0.001
	Prevention and screening of prostate cancer and other neoplasia in man health care	100.0_b_	81.1_c_	35.5_a_	0.001
	Care and rehabilitation of male urinary incontinence	70.0_b_	17.9_c_	3.2_a_	0.001
	Prevention and education on male sexual dysfunction	100.0_b_	42.5_c_	6.5_a_	0.001
	Guidelines on andropause in man health care	50.0_b_	15.1_a_	3.2_a_	0.001
	Early detection of breast cancer as a scheduled action for women's health	100.0_b_	82.1_c_	64.5_a_	0.006
	Identification and guidance of older adults’ caregivers	95.0_b_	44.3_c_	12.9_a_	0.001
	Evaluation of the caregiver's family functionality	90.0_b_	44.3_c_	9.7_a_	0.001
	Investigation of the caregiver's social support network	75.0_b_	34.9_c_	9.7_a_	0.001
	Informative support for caregivers	75.0_b_	55.7_b_	19.4_a_	0.001
	Evaluation of the caregiver’s stress	85.0_b_	26.4_c_	0.0_a_	0.001
	Support group for caregivers	30.0_b_	5.7_a_	3.2_a_	0.001
	Detection of violence against older adults by identifying symptoms	100_b_	72.6_c_	32.3_a_	0.001
	Detection of violence against older adults by listening to reports from other users	90.0_b_	74.5_b_	38.7_a_	0.001
	Detection of violence against older adults by their own declaration	90.0_b_	71.7_a.b_	54.8_a_	0.025
	Detection of violence against older adults by a case discussion	85.0_b_	40.6_c_	9.7_a_	0.001
	Detection of violence against older adults by team training	80.0_b_	24.5_c_	0.0_a_	0.001
	Detection of violence against older adults by home visits	100.0_b_	60.4_c_	32.3_a_	0.001
	Arraignment to the Social Assistance Reference Center (SARC) and the Specialized Social Assistance Reference Center (SSARC) after the identification of violence against older adults	95.0_b_	67.9_c_	35.5_a_	0.001
	Caregivers’ care and follow-up after the identification of violence against older adults	75.0_b_	34.0_c_	6.5_a_	0.001
	Compulsory notification after the identification of violence against older adults	55.0_b_	27.4_a_	35.5_a.b_	0.049
	Interdisciplinary care with service professionals after the identification of violence against older adults	85.0_b_	35.8_c_	12.9_a_	0.001
	Intersectoral follow-up of cases after the identification of violence against older adults	50.0_b_	7.5_a_	0.0_a_	0.001
d2	Scheduling periodic returns at the end of each care as a routine action for chronic noncommunicable diseases (CNCD)	100.0_b_	72.6_a_	54.8_a_	0.002
	Clarification and guidance of examination results as a routine action for CNCD	100.0_b_	90.6_b_	41.9_a_	0.001
	Prescription renewal without medical appointment as a routine action for CNCD	100.0_b_	80.2_c_	41.9_a_	0.001
	Active search for people with CNCD	85.0_b_	54.7_c_	12.9_a_	0.001
	Physical activities guidance as a routine action for CNCD	100.0_b_	81.1_c_	25.8_a_	0.001
	Conducting support groups for people with CNCD	90.0_b_	32.1_c_	0.0_a_	0.001
	Registration of patients at high risk as a routine action for CNCD	65.0_b_	33.0_c_	3.2_a_	0.001
	Referral to specialized service and its follow-up as a routine action for CNCD	90.0_b_	36.8_c_	9.7_a_	0.001
d2	Referral to adherent group in the service as a strategy to non-adherents with CNCD	30.0_b_	10.4_a_	6.5_a_	0.027
	Active search for non-adherents to the treatment with CNCD	85.0_b_	60.4_c_	25.8_a_	0.001
	Home visit as a strategy for non-adherents to the treatment with CNCD	95.0_b_	59.4_c_	19.4_a_	0.001
	Team discussion on approach alternatives as a strategy for non-adherents with CNCD	85.0_b_	37.7_c_	6.5_a_	0.001
	Discussion of cases of non-adherents with CNCD with external supervision	25.0_b_	11.3_b_	0.0_a_	0.019
	Convocation of missing adults with chronic diseases at risk of complications	90.0_b_	49.1_a_	38.7_a_	0.001
	Follow-up with protocol in the care of people with arterial hypertension	75.0_b_	44.3_c_	9.7_a_	0.001
	Three blood pressure measurements at different times in the care of people with arterial hypertension	90.0_b_	61.3_c_	35.5_a_	0.001
	Diet guidance in the care of people with arterial hypertension	100.0_b_	96.2_b_	74.2_a_	0.001
	Obesity treatment and/or prevention in the care of people with arterial hypertension	100.0_b_	67.9_c_	25.8_a_	0.001
	Periodic exam requests in the care of people with arterial hypertension	100.0_b_	86.8_b_	61.3_a_	0.001
	Introduction of non-drug therapy as a first alternative in the care of people with arterial hypertension	85.0_b_	56.6_c_	16.1_a_	0.001
	Cardiovascular risk assessment for drug therapy in the care of people with arterial hypertension	100.0_b_	57.5_c_	22.6_a_	0.001
	Group activities in the care of people with arterial hypertension	85.0_b_	50.9_c_	6.5_a_	0.001
	Guidance for physical activities in the care of people with arterial hypertension	100.0_b_	84.0_b_	45.2_a_	0.001
	Guidance and support for smoking cessation in the care of people with arterial hypertension	95.0_b_	64.2_c_	19.4_a_	0.001
	Investigation of alcohol and other drugs abuse in the care of people with arterial hypertension	85.0_b_	50.0_c_	0.0_a_	0.001
	Potassium as a routine examination for people with arterial hypertension	100.0_b_	92.5_b_	71.0_a_	0.001
	Triglycerides as a routine examination for people with arterial hypertension	100.0_a.b_	96.2_b_	83.9_a_	0.017
	Uric acid as a routine examination for people with arterial hypertension	100.0_b_	91.5_b_	77.4_a_	0.020
	Funduscopy as a routine examination for people with arterial hypertension	55.0_b_	18.9_c_	3.2_a_	0.001
	Dispensation of hydrochlorothiazide in the care of people with arterial hypertension	100.0_b_	86.8_b_	67.7_a_	0.004
	Dispensation of furosemide in the care of people with arterial hypertension	100.0_b_	86.8_b_	67.7_a_	0.004
	Dispensation of spironolactone in the care of people with arterial hypertension	95.0_b_	68.9_c_	48.4_a_	0.002
	Dispensation of atenolol in the care of people with arterial hypertension	65.0_a.b_	72.6_b_	45.2_a_	0.017
	Dispensation of propranolol in the care of people with arterial hypertension	100.0_b_	87.7_b_	58.1_a_	0.001
	Dispensation of methyldopa in the care of people with arterial hypertension	95.0_b_	79.2_b_	58.1_a_	0.006
	Dispensation of amlodipine besylate in the care of people with arterial hypertension	65.0_b_	59.4_b_	25.8_a_	0.002
	Dispensation of hydralazine hydrochloride in the care of people with arterial hypertension	20.0_b_	4.7%_a_	6.5_a.b_	0.049
	Dispensation of captopril in the care of people with arterial hypertension	100.0_b_	87.7_b_	64.5_a_	0.001
	Dispensation of enalapril in the care of people with arterial hypertension	100.0_b_	76.4_a_	64.5_a_	0.013
	Follow-up with protocol in the care of people with type 2 diabetes mellitus	75.0_b_	47.2_c_	16.1_a_	0.001
	Diet guidance for people with type 2 diabetes mellitus	100.0_b_	96.2_b_	80.6_a_	0.003
	Obesity treatment and/or prevention in the care of people with type 2 diabetes mellitus	95.0_b_	80.2_b_	32.3_a_	0.001
	Periodic exam requests in the care of people with type 2 diabetes mellitus	100.0_b_	90.6_b_	67.7_a_	0.001
	Control, evaluation and guidance of foot care for people with type 2 diabetes mellitus	100.0_b_	82.1_c_	41.9_a_	0.001
	Training for self-application of insulin in the care of people with type 2 diabetes mellitus	100.0_b_	82.1_c_	51.6_a_	0.001
	Glycometer supply in the care of people with type 2 diabetes mellitus	90.0_b_	75.5_b_	48.4_a_	0.002
	Group activities for people with type 2 diabetes mellitus	95.0_b_	52.8_c_	6.5_a_	0.001
	Physical activity guidance for people with type 2 diabetes mellitus	100.0_b_	84.0_b_	32.3_a_	0.001
	Guidance and support for smoking cessation for people with type 2 diabetes mellitus	90.0_b_	55.7_c_	12.9_a_	0.001
	Fasting blood sugar test as a routine examination for people with type 2 diabetes mellitus	100.0_a.b_	100.0_b_	87.1_a_	0.001
	Serum creatinine as a routine examination for people with type 2 diabetes mellitus	95.0_b_	86.8_b_	71.0_a_	0.039
	Glycated hemoglobin every three months, then every six months, as a routine examination for people with type 2 diabetes mellitus	95.0_b_	84.9_b_	54.8_a_	0.001
	Funduscopy as a routine examination for people with type 2 diabetes mellitus	75.0_b_	40.6_c_	6.5_a_	0.001
	Electrocardiography as a routine examination requested for people with type 2 diabetes mellitus	85.0_b_	66.0_a.b_	48.4_a_	0.026
d2	Dispensation of regular insulin in the care of people with type 2 diabetes mellitus	90.0_b_	77.4_b_	58.1_a_	0.024
	Dispensation of metformin in the care of people with type 2 diabetes mellitus	95.0_b_	79.2_b_	58.1_a_	0.006
	Dispensation of glibenclamide in the care of people with type 2 diabetes mellitus	100.0_b_	80.2_c_	54.8_a_	0.001
	Diagnosis and referrals to other levels of care as an action to bedridden people care	90.0_b_	62.3_c_	22.6_a_	0.001
	Periodic visits with support team as an action to bedridden people care	90.0_b_	54.7_c_	25.8_a_	0.001
	Periodic visits with physician as an action to bedridden people care	95.0_b_	61.3_a_	41.9_a_	0.001
	Home procedures as an action to bedridden people care	100.0_b_	84.0_b_	64.5_a_	0.004
	Oral hygiene guidelines as an action to bedridden people care	95.0_b_	55.7_c_	9.7_a_	0.001
	Home dental care as an action to bedridden people care	60.0_b_	17.9_c_	3.2_a_	0.001
	Guidelines on social rights as an action to bedridden people care	90.0_b_	30.2_c_	9.7_a_	0.001
	Prevention and follow-up in cases of alcohol and other drug use and abuse as an action to bedridden people care	85.0_b_	8.5_a_	0.0_a_	0.001
	Registration in medical records of domiciliary actions as an action to bedridden people care	100.0_b_	71.7_c_	22.6_a_	0.001
	Family care in case of death as an action to bedridden people care	95.0_b_	24.5_a_	12.9_a_	0.001
	Discussion of cases with health network as an action to bedridden people care	85.0_b_	39.6_c_	9.7_a_	0.001
d3	Vehicle for service use	75.0_b_	39.6_c_	19.4_a_	0.001
	Conducting electrocardiography in the service	80.0_b_	49.1_a_	38.7_a_	0.012
	Conducting rapid HIV test in the service	75.0_b_	49.1_c_	22.6_a_	0.001
	Older adults’ health as a theme of continuing training for the service team	55.0_b_	36.8_b_	6.5_a_	0.001
	Church–linked groups as a support or development of collaborative actions for older adults	30.0_b_	10.4_a_	6.5_a_	0.027
	SARC as a support for referrals and/or development of collaborative actions for older adults	90.0_b_	72.6_a.b_	58.1_a_	0.045
	SSARC as a support for referrals and/or development of collaborative actions for older adults	55.0_b_	31.1_a_	22.6_a_	0.047

Notes: Different letters represent statistically different values. p-value defined by the chi-square test.

In domain 2, we observe the smallest percentage differences of achievement between the quality groups. The smaller response percentages are among the indicators of domain 3, focused on equipment with which services can share actions and care directed to older adults and promote the team’s continued training on older adults’ health—for example, the Specialized Social Assistance Reference Center (SSARC).

Table 3 features the indicators with no significance according to the chi-square test. This includes 27% (42) out of the 155 indicators. Notably, most of them are in domain 3, in which non-differentiation between groups is due to the availability of infrastructure and, on the other hand, to the lack of support from the services network to older adults’ care in most services.

**Table 3:** Percentage distribution (%) of performance indicators frequencies in older adults’ health and aging care in primary care services, according to domain (d1, d2 and d3) and performance groups (G1, G2 and G3), with non-significant p-values, 2014 (n = 157).

Domain	Indicators	G1	G2	G3	p
d1	Mammography request every two years for women aged between 50 and 74 without risk factors or alteration of physical examination	50.0_a_	32.1_a_	32.3_a_	0.290
	Use of protocol as a strategy for detecting violence against older adults	20.0_b_	13.2_a.b_	3.2_a_	0.166
	Use of Dial 100 as a procedure performed after the identification of violence against older adults	20.0_a_	8.5_a_	6.5_a_	0.222
	Arraignment to the police as a procedure performed after the identification of violence against older adults	45.0_a_	24.5_a_	25.8_a_	0.166
	Referral to the family health support center or the support team as a procedure performed after the identification of violence against older adults	25.0_b_	17.0_a.b_	3.2_a_	0.075
d2	Blood pressure and/or blood glucose control at specific times and days and according to necessity as a routine action for chronic noncommunicable diseases (CNCD)	100.0_a_	90.6_a_	83.9_a_	0.160
	Referral to reference service as a strategy to non-adherents with CNCD	25.0_a_	26.4_a_	12.9_a_	0.293
	Discharge for non-adherents with CNCD	10.0_a_	6.6_a_	6.5_a_	0.854
	Call for missing people in activities and appointments scheduled for altered medical test results	73.7_a_	72.4_a_	67.7_a_	0.861
	Non-use of protocol in care for people with arterial hypertension	30.0_a_	38.7_a_	38.7_a_	0.755
	Urine analysis as a routine examination for people with arterial hypertension	100.0_a_	93.4_a_	83.9_a_	0.083
	Serum creatinine as a routine examination for people with arterial hypertension	100.0_a_	90.6_a_	83.9_a_	0.160
	Fasting blood sugar test as a routine examination for people with arterial hypertension	95.0_a_	95.3_a_	93.5_a_	0.928
	Total cholesterol and fractions (LDL and HDL) as a routine examination for people with arterial hypertension	100.0_a_	98.1_a_	96.8_a_	0.713
	Electrocardiography as a routine examination for people with arterial hypertension	100.0_a_	94.3_a_	83.9_a_	0.056
	Dispensation of mannitol in the care of people with arterial hypertension	15.0_b_	6.6_a.b_	0.0_a_	0.099
	Dispensation of metoprolol succinate in the care of people with arterial hypertension	20.0_a_	23.6_a_	22.6_a_	0.939
	Dispensation of verapamil hydrochloride in the care of people with arterial hypertension	30.0_a_	19.8_a_	22.6_a_	0.592
	Dispensation of sodium nitroprusside in the care of people with arterial hypertension	10.0_a_	6.6_a_	9.7_a_	0.778
	Non-use of protocol in the care of people with type 2 diabetes mellitus	25.0_a_	37.7_a_	38.7_a_	0.528
	Annual examination of total cholesterol and fractions and triglycerides in the care of people with type 2 diabetes mellitus	90.0_b_	69.8_a.b_	58.1_a_	0.052
	Urine analysis as a routine examination for people with type 2 diabetes mellitus	100.0_b_	91.5_a.b_	80.6_a_	0.058
	Microalbuminuria as a routine examination for people with type 2 diabetes mellitus	60.0_b_	50.0_b_	29.0_a_	0.056
	Total cholesterol and fractions (LDL and HDL) as a routine examination for people with type 2 diabetes mellitus	100.0_a_	92.5_a_	83.9_a_	0.111
	Triglycerides as a routine examination for people with type 2 diabetes mellitus	95.0_a_	92.5_a_	80.6_a_	0.110
	Dispensation of NPH insulin in the care of people with type 2 diabetes mellitus	90.0_a_	79.2_a_	67.7_a_	0.157
d3	Accessibility in service for people with disabilities (PWD) or with mobility impairments	85.0_a_	74.5_a_	61.3_a_	0.153
	Adapted restroom for people with disabilities or with mobility impairments	80.0_b_	53.8_a_	58.1%_a.b_	0.093
	Wheelchair in service	100.0_a_	89.6_a_	83.9_a_	0.177
	Application of DT vaccine in service	90.0_b_	78.3_a.b_	64.5_a_	0.093
	Application of influenza vaccine in service	90.0_b_	79.2_a.b_	64.5_a_	0.082
	Conduction of blood glucose test in service	100.0_a_	99.1_a_	93.5_a_	0.115
	Clinical laboratory tests collected in service	75.0_a_	67.0_a_	51.6_a_	0.174
	Access to the municipal or regional network of services for older adults’ care	45.0_b_	29.2_a.b_	16.1_a_	0.082
	Neighborhood associations as a support or development of collaborative actions for older adults	15.0_a_	5.7_a_	3.2_a_	0.212
	Senior living communities as a support for referrals and/or development of collaborative actions for older adults	20.0_a_	26.4_a_	12.9_a_	0.274
	Day-care center as a support for referrals and/or development of collaborative actions for older adults	5.0_a_	5.7_a_	6.5_a_	0.975
	Specialized service as a support for referrals and/or development of collaborative actions for older adults	10.0_a_	15.1_a_	6.5_a_	0.413
	Non-governmental organizations as a support for referrals and/or development of collaborative actions for older adults	10.0_a_	7.5_a_	3.2_a_	0.606
	Other services as a support for referrals and/or development of collaborative actions for older adults	20.0_b_	5.7_a_	12.9_a.b_	0.081

Notes: Different letters represent statistically different values. p-value defined by the chi-square test. Dial 100: service of arraignments and protection against human rights violations, available 24 hours every day.

Table 4 provides adjustments to the simple and multiple linear regression (SLR and MLR) models for the domain scores for the health planning and evaluation variables that remained in the final multiple model, although others may have been adjusted according to the criterion defined in the method. Analyzing the independent variables related to the differentiation of quality groups for domain 1 (d1), we found that the service typology, the consequences of evaluations, and changes induced by evaluations are distinctive to compose groups with different performance.

**Table 4:** Adjustments of simple and multiple linear regression (SLR and MLR) models for the domain scores by general planning activity variables and service evaluation and typology, based on the questionnaire for primary care services assessment and monitoring (QualiAB) 2014 (n = 157).

Domain	Variable	SLR	MLR			
	
		p-value	Coefficient			

			Beta	95%CI		p-value
d1 (r^2^ aj. = 0,82)	Traditional Basic Health Unit	< 0.001	0.198	20.590	-5.591	0.001
	Report on the problems identified for the municipal secretariat as the main unfolding of the evaluations	0.198	0.030	15.026	6.709	0.013
	The service lacked access to the evaluation results as the main unfolding of the evaluations	0.227	0.006	11.430	9.812	0.029
	Organization of assistance as evaluation–induced modification	0.238	0.052	45.379	8.615	0.036
	Management and organization of assistance as evaluation–induced modification	< 0.001	0.044	16.755	4.497	0.030
	Did not participate in any previous evaluation	0.001	0.003	-6.504	6.918	0.027
d2 (r^2^ aj. = 0,96)	Traditional Basic Health Unit	0.001	-0.051	-26.845	-4.549	0.004
	Non-use of epidemiological data for the service planning	0.017	-0.106	-14.023	1.282	0.006
	Organization of assistance as evaluation–induced modification	0.234	-0.024	-8.156	2.664	< 0.001
	Service management evaluation–induced modification	0.086	-0.030	-12.008	1.744	< 0.001
	Management and organization of assistance as evaluation–induced modification	< 0.001	-0.009	-7.612	5.322	< 0.001
	No evaluation–induced changes occurred in the service	0.077	-0.018	-18.964	14.800	< 0.001
	Did not participate in any previous evaluation	< 0.001	0.319	42.771	90.010	< 0.001
d3 (r^2^ aj. = 0,93)	Organization of assistance as evaluation–induced modification	0.247	3.627	18.534	63.012	< 0.001
	Service management evaluation–induced modification	0.043	2.729	8.451	52.981	0.007
	Management and organization of assistance as evaluation–induced modification	< 0.001	3.769	19.155	61.471	< 0.001
	No evaluation–induced changes occurred in the service	0.039	2.891	10.467	55.824	0.004
	Did not participate in any previous evaluation	0.007	2.944	10.756	54.840	0.004

Source: Questionnaire for primary care services assessment and monitoring (QualiAB) 2014 and the authors.

For domain 2 (d2), the independent variables that reflect relation with the differentiation of quality groups relate to the services typology, the use of epidemiological data, and the changes induced by evaluations, to form groups with better or worse performances. In domain 3 (d3), independent variables that influence on the formation of the different performance quality groups for the set of indicators evaluated relate to the changes induced by evaluations.

It is important to note the adjustment model (r^2^) for each domain proved to be great, a fact corroborated by its results: 0.82%, 0.96% and 0.93%, respectively.

## DISCUSSION

The results point to a better performance of PHC services in actions related to chronic noncommunicable diseases, matching with results from other studies^22 , 23^ and reflecting the undifferentiation with which services fulfill the scope of actions in older adults’ health and aging care. Prevention and promotion actions were incompletely incorporated into few services, and most of them lack a support network to collaborative work, resulting on the lack of comprehensive care for older adults’ health in the sample.

This is corroborated by a descriptive study previously conducted by the authors with analysis of the response frequencies of these primary care services on the indicators. This study identified: the presence of a support group for older adults’ caregivers in few services (8.2%), the use of a protocol to attend cases of violence against older adults (12.1%), a record with differentiated risk for people with chronic conditions (31.2%), and inclusion of older adults’ health as the subject of continuing education for the professional team (33.1%)^11^.

Aging and longevity are strongly associated with developing chronic diseases, because aging without any chronic disease is rare^24^. However, old age cannot be reduced to a set of illnesses in the actions developed in PHC.

The relevance of preventive actions for comprehensive care to the adult’s—and especially to the older adult’s—health apparently is a consensus; however, notably, practices tend to be restricted to the care of more prevalent CNCD^5 , 24^. It is obviously not a question of minimizing the importance of the quality of care for people with chronic diseases, but of emphasizing that older adults are often inserted in this set of problems, which does not automatically offer the necessary for them and for their aging.

Naturally, the actions performed by the HiperDia program, instituted since 2002 by the Brazilian Ministry of Health for people with systemic arterial hypertension and diabetes mellitus^25^, were implemented because chronic diseases represent one of the main demands for continuous follow-up in PHC services. However, to invest in improving the quality of this care is necessary, because the services fail to fully perform the recommended, as in relation to periodic funduscopy or foot care for people with diabetes, which occur in few services evaluated^11^.

In this study, it is possible to verify that the guidelines for health prevention and promotion are not being implemented in older adults and aging care, which also occurs with other stages of the life cycle and health demands^26 , 27^. In this sense, monitoring older adults’ functional capacity becomes a strategic indicator for health services^23^, especially for primary care services, which are potentially instrumentalized by the use of scales for functional and cognitive evaluation, among others, available in the Primary Care Guide no. 19^2^.

Studies on older adults’ care by primary care services conducted in Rio de Janeiro (RJ)^28^ and in Santos (SP)^29^ found a great disarticulation of the health services network in general and, especially, an absence of an older adults’ health care network, which hampers the supplying of this population health needs. Associations between the performance of the services in older adults’ care and the performance of activities indicative of local planning and evaluation show the importance of service management in determining the technical performance of programmatic activities. In particular, the low incorporation and use of data resulting from evaluation processes highlights the need to strengthen and improve monitoring and evaluation practices as subsidies for reprogramming technical actions of care, such as those directed to older adults’ health and to aging.

The limits of a cross-sectional study carried out in certain health regions and with a convenience sample should inhibit generalization of results. However, it brings significant elements in the face of the evaluation gap and the absence of instruments directed to assess older adults’ and aging care. In this sense, the study proved feasible to use an instrument that does not focus on this assessment but uses it as part of the diverse set of actions under the responsibility of PHC services.

It is necessary to advance in the provision of practices aimed at older adults by PHC services that seek to face the health demands of this population, aiming to understand the needs arising from aging and to collaborate so that individuals achieve old age with independence, autonomy and productivity^29^, because most chronic diseases that affect older adults have their main risk factor in their own age^24^. A contemporary model focused on aging, but especially in the older adult, needs to gather a sequence of education, health promotion, prevention of evitable diseases and postponement of injuries. To achieve positive results, an articulated, referenced network with information system built is essential^24 , 30^.

## CONCLUSIONS

The performance of comprehensive care for older adults in primary care services in the Brazilian Unified Health System in the state of São Paulo is incipient. Incipience is stronger in healthy aging care activities, while those for treatment of some chronic diseases show relatively better performance.

To distinguish three groups of decreasing performance services was possible according to the three domains evaluated. The intermediate group, group 2, concentrates most services (67%). The best group, group 1, is minority (12%). The worst performing group, group 3, concentrates 31 services (20%) and shows worst performance in all domains, especially in the healthy aging care.

Results indicate the need to improve older adults’ care in all domains, emphasizing, however, the urgency of improving local performance and health network in the effective implementation of actions to aging and older adults’ care according to the Ministry of Health guidelines. The precariousness of performance of various services also draws attention.

The evaluation framework was able to characterize and differentiate the current performance of older adults’ care in PHC services and can be used to establish and disseminate new normative standards for PHC.
