# Supplementary material for: Evaluation of comprehensive care for older adults in primary care services
Source: Rev Saude Publica. 2020 Jan 14;54:6. doi: 10.11606/s1518-8787.2020054001370 (PMC6961969; doi:10.11606/s1518-8787.2020054001370)
Supplement: Translation

# Translation: Avaliação da atenção integral ao idoso em serviços de atenção primária

**Authors:** Nádia Placideli, Elen Rose Lodeiro Castanheira, Adriano Dias, Pedro Alcântara da Silva, Josiane Lozigia Fernandes Carrapato, Patricia Rodrigues Sanine, Dinair Ferreira Machado, Carolina Siqueira Mendonça, Thais Fernanda Tortorelli Zarili, Luceime Olivia Nunes, José Fernando Casquel Monti, Zulmira Maria de Araújo Hartz, Maria Ines Battistella Nemes

**Affiliations:** I Universidade Estadual Paulista. Faculdade de Medicina de Botucatu. Departamento de Enfermagem. Botucatu, SP, Brasil; II Universidade Estadual Paulista. Faculdade de Medicina de Botucatu. Programa de Pós-Graduação em Saúde Coletiva. Departamento de Saúde Pública. Botucatu, SP, Brasil; III Universidade de Lisboa. Instituto de Ciências Sociais. Lisboa. Portugal; IV Instituto Toledo de Ensino. Bauru, SP, Brasil; V >Universidade Federal de São Carlos. Faculdade de Medicina. São Carlos, SP, Brasil; VI Universidade Nova de Lisboa. Instituto de Higiene e Medicina Tropical. Lisboa. Portugal; VII Universidade de São Paulo. Faculdade de Medicina. Departamento de Medicina Preventiva. São Paulo, SP, Brasil

**Keywords:** Serviços de Saúde para Idosos, Assistência Integral à Saúde, Atenção Primária à Saúde, Pesquisa sobre Serviços de Saúde, Sistema Único de Saúde

## Abstract

**OBJETIVO:**

Avaliar o desempenho da atenção integral ao idoso em serviços de atenção primária do Sistema Único de Saúde no estado de São Paulo, Brasi

**lMÉTODOS:**

Um total de 157 serviços de atenção primária de cinco regiões de saúde do centro-oeste paulista respondeu, de outubro a dezembro de 2014, o instrumento pré-validado Questionário de Avaliação e Monitoramento de Serviços de Atenção Básica 2014. Foram selecionadas 155 questões, com base nas políticas e diretrizes nacionais sobre essa temática. As respostas indicam o desempenho do serviço na atenção ao idoso, agrupadas em três domínios de análise: atenção à saúde para o envelhecimento ativo e saudável (45 indicadores, d1), atenção às doenças crônicas não transmissíveis (89 indicadores, d2) e rede de apoio na atenção ao envelhecimento (21 indicadores, d3). A medida de desempenho foi a soma de respostas positivas (valor 1) ou negativas (valor 0) para cada indicador. Os serviços foram agrupados segundo k-médias dos escores de desempenho de cada um dos domínios. Após a ponderação dos domínios (testes Z), foram estimadas as associações entre os escores de cada domínio e variáveis independentes de gestão (tipologia, planejamento e avaliação dos serviços), por meio de regressão linear simples e múltipla.

**RESULTADOS:**

A atenção às doenças crônicas não transmissíveis (d2) mostrou, para todos os agrupamentos, melhor desempenho médio (55,7) do que os domínios d1 (35,4) e d3 (39,2). O desempenho do serviço na área geral de planejamento e avaliação esteve associado ao desempenho da atenção ao idoso.

**CONCLUSÕES:**

Os serviços avaliados apresentaram implementação incipiente da atenção integral ao idoso. O quadro avaliativo pode contribuir para processos de melhoria da qualidade da atenção primária à saúde.

## INTRODUÇÃO

A atenção primária à saúde (APS) tem sido apontada como nível prioritário para assistir e monitorar o estado de saúde da população idosa, além de atuar na prevenção de agravos e promoção da saúde em busca do envelhecimento saudável. Nessa perspectiva, a Organização Mundial de Saúde (OMS) propôs em 2004 que os serviços de atenção primária deveriam adaptar-se para atender aos idosos adequadamente^1^. No Brasil, diante do fenômeno do envelhecimento populacional, o Ministério da Saúde publicou em 2007 o *Caderno de Atenção Básica* nº 19, com orientações às equipes de atenção primária para uma maior resolubilidade das demandas dos idosos^2^.

A expansão da rede pública de APS no país, associada à necessidade de fortalecimento das estruturas de gestão e de redefinição dos papéis nas três esferas de governo, impulsionou a institucionalização de mecanismos de pactuação e avaliação, objetivando auxiliar o processo decisório e melhorar o desempenho do sistema público de saúde^3 , 4^. Especialmente na última década, estratégias de avaliação e monitoramento passaram a ser reconhecidas como ferramentas essenciais à mensuração da efetividade dos sistemas de saúde em todos os níveis de atenção^4^.

Nesse contexto, cresce a importância de avaliar os serviços para a atenção à saúde do idoso, especialmente na APS, a fim de conhecer os avanços na implementação das recomendações sintetizadas no *Caderno de Atenção Básica* nº 19^2^, que abrangem, em linhas gerais, ações de promoção e prevenção para o alcance do envelhecimento ativo e saudável, gestão das doenças crônicas não transmissíveis (DCNT) e o trabalho em rede.

Estudos nacionais e internacionais realizados na perspectiva de avaliar a atenção prestada aos idosos na APS assinalam a necessidade de ampliação de práticas preventivas e de promoção da saúde, pouco frequentes e com baixa diversidade de ações, além da importância da construção de redes de atenção integral à saúde do idoso, ordenadas pelos serviços de APS^5^. O instrumento QualiAB 2014 (Questionário de Avaliação e Monitoramento de Serviços de Atenção Básica) propõe, entre seus indicadores para a avaliação abrangente dos serviços de atenção primária, os direcionados à atenção ao idoso^11^.

Apesar das proposições políticas e tecnológicas disponíveis no país para a atuação da APS nesse segmento, ainda é escassa a literatura sobre as ações efetivamente implantadas^5^avaliando sua organização e oferta, bem como sobre os desafios gerados pelo envelhecimento populacional para esses serviços^5^. A maioria dos estudos avalia as ações desenvolvidas na APS segundo a percepção dos idosos sobre cuidados recebidos, sendo raros aqueles que tomam como foco avaliativo a organização de ações de forma abrangente e direcionada ao trabalho realizado pelas equipes.

O presente estudo tem por objetivo avaliar o desempenho da atenção integral ao idoso em serviços de atenção primária à saúde segundo seus gerentes e profissionais, assim como analisar a relação entre o desempenho e indicadores de planejamento e avaliação em saúde.

## MÉTODOS

Pesquisa avaliativa, de corte transversal, baseada na análise dos resultados da aplicação do QualiAB em 2014. A avaliação ocorreu em serviços de APS localizados em cinco regiões de saúde do centro-oeste paulista: Bauru, Jaú, Lins, Polo Cuesta e Vale do Jurumirim, totalizando 68 municípios e 303 serviços, segundo o CNES^12^(excluindo-se cadastros duplicados e não correspondentes a serviços de APS).

Trata-se de uma amostra por conveniência, constituída por cinco regiões de saúde escolhidas para uma aplicação do tipo censitária do instrumento. As regiões escolhidas localizam-se em áreas próximas à instituição de ensino superior responsável pela pesquisa, com as quais existem parcerias anteriores em projetos de apoio à gestão e avaliação de serviços de APS utilizando o mesmo instrumento^13^. Houve adesão de 157 serviços distribuídos em 41 municípios, representando 63% do total existente no período avaliado^12^. A adesão foi voluntária, uma vez que não há nenhuma forma de incentivo financeiro ou premiação no processo.

O QualiAB 2014 foi respondido on-line pelas equipes de cada serviço de saúde, após adesão do gestor municipal e dos gerentes ou responsáveis de cada serviço. Essa aplicação integrou o processo de atualização e revalidação do instrumento, originalmente validado em 2007^13^.

Essa versão do QualiAB é composta por 126 questões de múltipla escolha, que geram indicadores compostos para avaliação global dos serviços de APS, de modo a abranger o conjunto diversificado de ações de saúde atribuídas à atenção primária, conforme previsto na Política Nacional de Atenção Básica (PNAB)^16^. Trata-se de um questionário desenvolvido para avaliar e monitorar os serviços quanto à estrutura e organização do processo de trabalho por meio de indicadores de assistência e gerenciamento, independentemente do tipo de unidade, ou seja, tanto organizada segundo a Estratégia Saúde da Família quanto por outro tipo de arranjo entre as várias composições existentes^13^.

Para o presente estudo, foram eleitas e categorizadas as variáveis do QualiAB 2014 relacionadas com a atenção à saúde do idoso e envelhecimento, definindo-se assim 155 indicadores de desempenho. Primeiramente foram identificados e analisados documentos sobre esse objeto de avaliação, para possibilitar sua delimitação a fim de construir um modelo lógico-teórico que orientasse a seleção dos indicadores e sua organização em domínios. Embora não esteja instituído um programa delineado especificamente para a saúde do idoso, como ocorre com outros segmentos, existem diretrizes e normas técnicas que permitem delimitar as ações que devem ser desenvolvidas nesse nível de atenção^17^.

Os principais documentos utilizados foram a Política Nacional de Saúde da Pessoa Idosa^18^e as diretrizes do Ministério da Saúde publicadas no *Caderno de Atenção Básica* nº 19^2^, no Plano de Ações Estratégicas para o Enfrentamento das Doenças Crônicas Não Transmissíveis no Brasil 2011–2022^19^e no manual técnico *Diretrizes para o cuidado das pessoas com doenças crônicas nas redes de atenção à saúde e nas linhas de cuidado prioritárias* ^20^.

Os indicadores selecionados do QualiAB 2014 foram reunidos segundo três grandes conjuntos de atributos considerados essenciais à atenção integral à saúde do idoso e ao envelhecimento, na forma de três domínios: atenção à saúde para o envelhecimento ativo e saudável (d1, composto por 45 indicadores), atenção às doenças crônicas não transmissíveis (DCNT) (d2, composto por 89 indicadores) e estrutura e rede de apoio na atenção ao envelhecimento (d3, composto por 21 indicadores).

A análise estatística foi conduzida a partir de diferentes estratégias. A cada um dos domínios foi atribuído um escore final equivalente à soma de respostas dicotômicas aos indicadores que o compõe, sendo que o valor 1 se refere à realização e valor 0 à não realização. Para cada domínio, foram definidos três *clusters* (grupos de desempenho) por meio de k-médias relativas aos escores obtidos, de modo a alcançar a máxima heterogeneidade possível entre os diferentes grupos e a máxima homogeneidade interna em cada grupo^21^. Os três grupos foram ordenados por gradação decrescente das k-médias obtidas pelos serviços, denominando-se grupo 1 (G1) o dos serviços com maiores k-médias e grupo 3 (G3) o dos com menores k-médias.

A segunda etapa da análise testou a associação da composição dos grupos com as respostas a 30 indicadores do QualiAB 2014 que caracterizam as atividades relacionadas ao planejamento e avaliação geral do serviço, mostradas no Quadro 1 Foram estimadas as associações entre os indicadores selecionados e cada um dos três domínios por meio de testes do qui-quadrado seguidos por testes Z. Como cada domínio foi composto por quantidades diferentes de indicadores (com escores máximos de 45, 89 e 21, respectivamente), os escores foram padronizados, a fim de deixá-los dentro de uma mesma escala de magnitude.

**Quadro 1:** Variáveis independentes relativas às dimensões de planejamento e avaliação utilizadas nos modelos de regressão linear simples e múltipla, com base no Questionário de Avaliação e Monitoramento de Serviços de Atenção Básica 2014.

Dimensão	Nucleos temáticos	Variáveis independentes
Planejamento	Tipo de serviço	- Unidade de saúde da família (USF) - Unidade básica de saúde (UBS) tradicional - UBS com Estratégia Saúde da Família (ESF) - UBS com Programa de Agentes Comunitários de Saúde (PACS) - UBS ou USF integrada a uma unidade de pronto atendimento - Posto avançado de saúde (PAS)
	Localização geográfica	- Rural - Urbana central - Urbana periférica
	Planejamento da área de abrangência do serviço	- Área definida por planejamento participativo, considerando a realidade local e facilidade de acesso - Área definida pela equipe do serviço - Não há definição da área de abrangência
	Apoio matricial	- Núcleo de apoio à saúde da família (NASF) - Equipe multiprofissional - Não é realizado por nenhuma instância
Avaliação	Uitilização de dados epidemiológicos para planejamento	- Pela gestão municipal e unidade, para planejamento - Pela gestão municipal, mas não são apropriados pela unidade - Não são utilizados
	Participação em avaliações nos últimos três anos	- Participou - Não participou
	Desdobramentos a partir das avaliações realizadas	- Plano anual de trabalho da equipe - Planejamento e reprogramação das atividades com participação da equipe multiprofissional - Realização de relatório dos problemas identificados para secretaria municipal - O serviço não teve acesso aos resultados - Não foi realizada avaliação
	Mudanças induzidas por avaliações	- Organização da assistência - Gerenciamento do serviço - Gerenciamento e organização da assistência - Não houve mudanças - Não participou de nenhuma avaliação anterior

Com os escores padronizados, foram ajustados modelos de regressão linear simples e múltipla, tendo como variáveis independentes as características do serviço selecionadas ( Quadro 1 ) e como variáveis resposta os escores de cada domínio. As variáveis que obtiveram valores de r^2^acima de 50% e níveis de significância do modelo menores que 0,25 com distribuição normal dos resíduos nos ajustes simples foram levadas ao ajuste múltiplo, no qual se mantiveram apenas aquelas com r^2^acima de 80%, valores p < 0,05 e distribuição normal dos resíduos. Todos os passos da análise estatística foram desenvolvidos utilizando o software SPSS versão 20.0.

Este estudo foi aprovado pelo Comitê de Ética em Pesquisa da Faculdade de Medicina de Botucatu, Universidade Estadual Paulista por meio do parecer nº 855.404.

## RESULTADOS

Entre os 157 serviços de APS avaliados, segundo autoclassificação, prevaleceram as unidades de saúde da família (USF; 42%), seguidas pelas unidades básicas de saúde (UBS) tradicionais (36,9%). Foi observada a presença da Estratégia Saúde da Família (ESF) em 8,2% das UBS e do Programa de Agentes Comunitários de Saúde (PACS) em 8,9%; 1,2% eram UBS tradicionais ou USF integradas a pronto atendimento, e 2,5% dos serviços optaram por classificar-se como outras modalidades.

Os 45 indicadores do instrumento QualiAB 2014 eleitos para o domínio 1 (d1) abarcam as ações de promoção, prevenção e assistência relacionadas diretamente à pessoa idosa; atenção ao envelhecimento na prevenção de agravos e promoção da saúde; estratégias e ações em situação de violência contra a pessoa idosa; e atenção aos cuidadores de idosos. O domínio 2 (d2) agrupou 89 indicadores correspondentes às ações de rotina para pessoas com DCNT e estratégias de abordagem à não adesão ao tratamento; atenção programática, exames de rotina e medicamentos disponíveis para pessoas com hipertensão arterial e para pessoas com diabetes mellitus tipo II; e ações para o cuidado de pessoas acamadas. No domínio 3 (d3) foram agrupados 21 indicadores relacionados a infraestrutura, insumos e qualificação profissional que viabilizam a prevenção e a promoção da saúde na busca pelo envelhecimento ativo e saudável, além da rede de serviços para atenção à saúde do idoso no trabalho conjunto com a atenção primária. A distribuição dos serviços nos diferentes grupos (G1, G2 e G3) e o desempenho médio conforme cada domínio de análise pode ser observado na Tabela 1 .

**Tabela 1:** Desempenho dos serviços de atenção primária à saúde do idoso e ao envelhecimento, agrupados segundo k-médias, por domínio de análise, 2014 (n = 157).

Domínios	Grupos	N	Média	DP	Mínimo	Máximo
Atenção à saúde para o envelhecimento ativo e saudável (d1)	1	20	68,4	9,0	47,5	83,1
	2	106	35,4	13,9	5,9	71,2
	3	31	12,0	5,3	1,9	23,7
Atenção às doenças crônicas não transmissíveis (d2)	1	19	73,6	3,8	66,0	80,0
	2	105	55,7	8,0	37,0	71,0
	3	31	35,4	7,5	20,0	49,0
Estrutura e rede de apoio na atenção ao envelhecimento (d3)	1	20	51,0	11,1	33,9	67,8
	2	106	39,2	10,1	12,7	59,3
	3	31	30,2	8,8	12,7	50,8

DP: desvio-padrão

Chama atenção que nos três domínios a maior parte dos serviços concentra-se no grupo 2 de desempenho, ainda que com variações entre as médias alcançadas. Os maiores valores foram obtidos no domínio de atenção às DCNT (d2), no qual também se observam as maiores médias.

No domínio 1 destaca-se que os serviços apresentaram as menores médias entre os grupos de menor desempenho. Quanto ao domínio 3, ressalta-se que o grupo 1 obteve nele sua menor média (51,0) dentre todos os domínios. Chama a atenção que a maioria dos serviços realiza menos da metade do conjunto de indicadores avaliados, compondo os grupos 2 (39,2; d2) e o 3 (30,2; d3). Vale destacar a menor diferença entre as médias dos grupos neste domínio, o que sugere que os serviços apresentam menor diferenciação em relação aos indicadores avaliados.

Nas tabelas 2 e 3 são apresentadas as frequências dos indicadores por domínio e por grupo de desempenho (G1, G2 e G3), permitindo aprofundar a compreensão das medidas de tendência central. As diferenças entre os grupos são significativas em 72,9% (113) dos 155 indicadores avaliados, apresentados na Tabela 2 . Os serviços pertencentes ao G1 apresentam altos percentuais de realização da maior parte das ações avaliadas; no entanto, mesmo eles não contemplam a totalidade para uma atenção integral à saúde do idoso e ao envelhecimento.

**Tabela 2:** Distribuição percentual (%) das frequências dos indicadores de desempenho na atenção à saúde do idoso e ao envelhecimento em serviços de atenção primária, segundo domínio (d1, d2 e d3) e grupos de desempenho (G1, G2 e G3), com p-valores significativos, 2014 (n = 157).

Domínio	Indicadores	G1	G2	G3	p
d1	Avaliação cognitiva	90,0_b_	38,7_c_	9,7_a_	0,001
	Avaliação de quadros depressivos	90,0_b_	43,4_c_	3,2_a_	0,001
	Avaliação funcional	85,0_b_	19,8_c_	0,0_a_	0,001
	Orientações para prevenção de quedas	95,0_b_	60,4_c_	0,0_a_	0,001
	Orientações sobre alimentação e nutrição	100,0_b_	72,6_c_	6,5_a_	0,001
	Orientações sobre incontinência urinária	95,0_b_	48,1_c_	0,0_a_	0,001
	Orientações sobre a importância da atividade física	95,0_b_	42,5_c_	0,0_a_	0,001
	Ações quanto à sexualidade e doenças sexualmente transmissíveis (DST)	90,0_b_	32,1_c_	0,0_a_	0,001
	Orientações sobre menopausa e andropausa	95,0_b_	44,3_c_	6,5_a_	0,001
	Orientações sobre saúde bucal	95,0_b_	68,9_c_	16,1_a_	0,001
	Orientações sobre direitos do idoso	70,0_b_	29,2_c_	3,2_a_	0,001
	Orientações quanto à violência contra idosos	85,0_b_	32,1_c_	0,0_a_	0,001
	Investigação do uso abusivo de álcool e outras drogas	75,0_b_	23,6_c_	0,0_a_	0,001
	Atenção domiciliar	100,0_b_	59,4_a_	29,0_a_	0,001
	Apoio a idosos em instituições de longa permanência para idosos (ILPI)	50,0_b_	15,1_b_	9,7_a_	0,001
	Atendimento odontológico para idosos	75,0_b_	52,8_a_	25,8_a_	0,002
	Educação em saúde na comunidade sobre promoção do envelhecimento saudável	55,0_b_	21,7_c_	3,2_a_	0,001
	Educação em saúde no serviço sobre promoção do envelhecimento saudável	80,0_b_	22,6_c_	0,0_a_	0,001
	Prevenção e rastreamento de câncer de próstata e outras neoplasias na atenção à saúde do homem	100,0_b_	81,1_c_	35,5_a_	0,001
	Cuidados e reabilitação da incontinência urinária masculina	70,0_b_	17,9_c_	3,2_a_	0,001
	Prevenção e educação sobre impotência sexual masculina	100,0_b_	42,5_c_	6,5_a_	0,001
	Orientações sobre andropausa na atenção à saúde do homem	50,0_b_	15,1_a_	3,2_a_	0,001
	Detecção precoce de câncer de mama como ação programada à saúde da mulher	100,0_b_	82,1_c_	64,5_a_	0,006
	Identificação e orientação de cuidadores de idosos	95,0_b_	44,3_c_	12,9_a_	0,001
	Avaliação da funcionalidade familiar do cuidador	90,0_b_	44,3_c_	9,7_a_	0,001
	Investigação da rede de apoio social do cuidador	75,0_b_	34,9_c_	9,7_a_	0,001
	Suporte informativo aos cuidadores	75,0_b_	55,7_b_	19,4_a_	0,001
	Avaliação do estresse do cuidador	85,0_b_	26,4_c_	0,0_a_	0,001
	Grupo de apoio aos cuidadores	30,0_b_	5,7_a_	3,2_a_	0,001
	Detecção de violência contra idosos por meio de identificação de sintomas	100_b_	72,6_c_	32,3_a_	0,001
	Detecção de violência contra idosos por meio de escuta de relatos de outros usuários	90,0_b_	74,5_b_	38,7_a_	0,001
	Detecção de violência contra idosos por meio de declaração do próprio idoso	90,0_b_	71,7_a,b_	54,8_a_	0,025
	Detecção de violência contra idosos por meio de discussão de caso	85,0_b_	40,6_c_	9,7_a_	0,001
	Detecção de violência contra idosos por meio de realização de capacitação da equipe	80,0_b_	24,5_c_	0,0_a_	0,001
	Detecção de violência contra idosos por meio de visita domiciliar	100,0_b_	60,4_c_	32,3_a_	0,001
	Denúncia ao Centro de Referência de Assistência Social (CRAS) e ao Centro de Referência Especializado de Assistência Social (CREAS) após a identificação de violência contra idosos	95,0_b_	67,9_c_	35,5_a_	0,001
	Atendimento e acompanhamento dos cuidadores após a identificação de violência contra idosos	75,0_b_	34,0_c_	6,5_a_	0,001
	Notificação compulsória após a identificação de violência contra idosos	55,0_b_	27,4_a_	35,5_a,b_	0,049
	Atendimento interdisciplinar com profissionais do serviço após a identificação de violência contra idosos	85,0_b_	35,8_c_	12,9_a_	0,001
	Acompanhamento intersetorial dos casos após a identificação de violência contra idosos	50,0_b_	7,5_a_	0,0_a_	0,001
d2	Agendamento de retornos peródicos ao final de cada atendimento como ação de rotina para doenças crônicas não transmissíveis (DCNT)	100,0_b_	72,6_a_	54,8_a_	0,002
	Esclarecimento e orientação dos resultados de exames como ação de rotina para DCNT	100,0_b_	90,6_b_	41,9_a_	0,001
	Renovação de receitas sem consulta médica no dia como ação de rotina para DCNT	100,0_b_	80,2_c_	41,9_a_	0,001
	Busca ativa de pessoas com DCNT	85,0_b_	54,7_c_	12,9_a_	0,001
	Orientação de atividades físicas como ação de rotina para DCNT	100,0_b_	81,1_c_	25,8_a_	0,001
	Realização de grupos de apoio para pessoas com DCNT	90,0_b_	32,1_c_	0,0_a_	0,001
	Registro dos pacientes de risco diferenciado como ação de rotina para DCNT	65,0_b_	33,0_c_	3,2_a_	0,001
	Encaminhamento para serviço especializado e seguimento pelo próprio serviço como ação de rotina para DCNT	90,0_b_	36,8_c_	9,7_a_	0,001
d2	Encaminhamento para grupo de adesão no próprio serviço como estratégia aos não aderentes com DCNT	30,0_b_	10,4_a_	6,5_a_	0,027
	Busca ativa dos não aderentes ao tratamento com DCNT	85,0_b_	60,4_c_	25,8_a_	0,001
	Visita domiciliar como estratégia aos não aderentes ao tratamento com DCNT	95,0_b_	59,4_c_	19,4_a_	0,001
	Discussão em equipe sobre alternativas de abordagem como estratégia aos não aderentes com DCNT	85,0_b_	37,7_c_	6,5_a_	0,001
	Discussão dos casos dos não aderentes com DCNT com supervisão externa	25,0_b_	11,3_b_	0,0_a_	0,019
	Convocação de adultos com doenças crônicas faltosos com risco de complicações	90,0_b_	49,1_a_	38,7_a_	0,001
	Acompanhamento com protocolo na atenção às pessoas com hipertensão arterial	75,0_b_	44,3_c_	9,7_a_	0,001
	Aferição de três medidas de pressão arterial em momentos diferentes na atenção às pessoas com hipertensão arterial	90,0_b_	61,3_c_	35,5_a_	0,001
	Orientação de dieta na atenção às pessoas com hipertensão arterial	100,0_b_	96,2_b_	74,2_a_	0,001
	Tratamento e/ou prevenção de obesidade na atenção às pessoas com hipertensão arterial	100,0_b_	67,9_c_	25,8_a_	0,001
	Solicitação periódica de exames na atenção às pessoas com hipertensão arterial	100,0_b_	86,8_b_	61,3_a_	0,001
	Introdução de terapêutica não medicamentosa como primeira alternativa na atenção às pessoas com hipertensão arterial	85,0_b_	56,6_c_	16,1_a_	0,001
	Avaliação de risco cardiovascular para terapêutica medicamentosa na atenção às pessoas com hipertensão arterial	100,0_b_	57,5_c_	22,6_a_	0,001
	Atividades em grupo na atenção às pessoas com hipertensão arterial	85,0_b_	50,9_c_	6,5_a_	0,001
	Orientação de atividade física na atenção às pessoas com hipertensão arterial	100,0_b_	84,0_b_	45,2_a_	0,001
	Orientação e suporte para abandono do tabagismo na atenção às pessoas com hipertensão arterial	95,0_b_	64,2_c_	19,4_a_	0,001
	Investigação de uso abusivo de álcool e outras drogas na atenção às pessoas com hipertensão arterial	85,0_b_	50,0_c_	0,0_a_	0,001
	Potássio como exame de rotina para pessoas com hipertensão arterial	100,0_b_	92,5_b_	71,0_a_	0,001
	Triglicerídeos como exame de rotina para pessoas com hipertensão arterial	100,0_a,b_	96,2_b_	83,9_a_	0,017
	Ácido úrico como exame de rotina para pessoas com hipertensão arterial	100,0_b_	91,5_b_	77,4_a_	0,020
	Exame de fundo de olho como exame de rotina para pessoas com hipertensão arterial	55,0_b_	18,9_c_	3,2_a_	0,001
	Dispensação de hidroclorotiazida na atenção às pessoas com hipertensão arterial	100,0_b_	86,8_b_	67,7_a_	0,004
	Dispensação de furosemida na atenção às pessoas com hipertensão arterial	100,0_b_	86,8_b_	67,7_a_	0,004
	Dispensação de espironolactona na atenção às pessoas com hipertensão arterial	95,0_b_	68,9_c_	48,4_a_	0,002
	Dispensação de atenolol na atenção às pessoas com hipertensão arterial	65,0_a,b_	72,6_b_	45,2_a_	0,017
	Dispensação de propanolol na atenção às pessoas com hipertensão arterial	100,0_b_	87,7_b_	58,1_a_	0,001
	Dispensação de metildopa na atenção às pessoas com hipertensão arterial	95,0_b_	79,2_b_	58,1_a_	0,006
	Dispensação de besilato de anlodipino na atenção às pessoas com hipertensão arterial	65,0_b_	59,4_b_	25,8_a_	0,002
	Dispensação de cloridrato de hidralazina na atenção às pessoas com hipertensão arterial	20,0_b_	4,7%_a_	6,5_a,b_	0,049
	Dispensação de captopril na atenção às pessoas com hipertensão arterial	100,0_b_	87,7_b_	64,5_a_	0,001
	Dispensação de enalapril na atenção às pessoas com hipertensão arterial	100,0_b_	76,4_a_	64,5_a_	0,013
	Acompanhamento com protocolo na atenção às pessoas com diabetes mellitus tipo II	75,0_b_	47,2_c_	16,1_a_	0,001
	Orientação de dieta às pessoas com diabetes mellitus tipo II	100,0_b_	96,2_b_	80,6_a_	0,003
	Tratamento e/ou prevenção de obesidade na atenção às pessoas com diabetes mellitus tipo II	95,0_b_	80,2_b_	32,3_a_	0,001
	Solicitação periódica de exames na atenção às pessoas com diabetes mellitus tipo II	100,0_b_	90,6_b_	67,7_a_	0,001
	Controle, avaliação e orientação de cuidados com os pés na atenção às pessoas com diabetes mellitus tipo II	100,0_b_	82,1_c_	41,9_a_	0,001
	Treinamento para autoaplicação de insulina na atenção às pessoas com diabetes mellitus tipo II	100,0_b_	82,1_c_	51,6_a_	0,001
	Fornecimento do glicosímetro na atenção às pessoas com diabetes mellitus tipo II	90,0_b_	75,5_b_	48,4_a_	0,002
	Atividades em grupo para pessoas com diabetes mellitus tipo II	95,0_b_	52,8_c_	6,5_a_	0,001
	Orientação de atividade física para pessoas com diabetes mellitus tipo II	100,0_b_	84,0_b_	32,3_a_	0,001
	Orientação e suporte para abandono do tabagismo para pessoas com diabetes mellitus tipo II	90,0_b_	55,7_c_	12,9_a_	0,001
	Glicemia de jejum como exame de rotina para pessoas com diabetes mellitus tipo II	100,0_a,b_	100,0_b_	87,1_a_	0,001
	Creatinina sérica como exame de rotina para pessoas com diabetes mellitus tipo II	95,0_b_	86,8_b_	71,0_a_	0,039
	Hemoglobina glicada a cada três meses, depois a cada seis meses, como exame de rotina para pessoas com diabetes mellitus tipo II	95,0_b_	84,9_b_	54,8_a_	0,001
	Exame de fundo de olhos como exame de rotina para pessoas com diabetes mellitus tipo II	75,0_b_	40,6_c_	6,5_a_	0,001
	Eletrocardiograma como exame solicitado de rotina para pessoas com diabetes mellitus tipo II	85,0_b_	66,0_a,b_	48,4_a_	0,026
d2	Dispensação de insulina regular na atenção às pessoas com diabetes mellitus tipo II	90,0_b_	77,4_b_	58,1_a_	0,024
	Dispensação de metformina na atenção às pessoas com diabetes mellitus tipo II	95,0_b_	79,2_b_	58,1_a_	0,006
	Dispensação de glibenclamida na atenção às pessoas com diabetes mellitus tipo II	100,0_b_	80,2_c_	54,8_a_	0,001
	Diagnóstico e encaminhamentos para outros níveis de atenção como ação ao cuidado de pessoas acamadas	90,0_b_	62,3_c_	22,6_a_	0,001
	Visitas periódicas com equipe de apoio como ação ao cuidado de pessoas acamadas	90,0_b_	54,7_c_	25,8_a_	0,001
	Visitas periódicas com médico como ação ao cuidado de pessoas acamadas	95,0_b_	61,3_a_	41,9_a_	0,001
	Procedimentos domiciliares como ação ao cuidado de pessoas acamadas	100,0_b_	84,0_b_	64,5_a_	0,004
	Orientações de higiene bucal como ação ao cuidado de pessoas acamadas	95,0_b_	55,7_c_	9,7_a_	0,001
	Assistência odontológica domiciliar como ação ao cuidado de pessoas acamadas	60,0_b_	17,9_c_	3,2_a_	0,001
	Orientações quanto aos direitos sociais como ação ao cuidado de pessoas acamadas	90,0_b_	30,2_c_	9,7_a_	0,001
	Prevenção e acompanhamento em casos de uso e abuso de álcool e outras drogas como ação ao cuidado de pessoas acamadas	85,0_b_	8,5_a_	0,0_a_	0,001
	Registro das ações domiciliares em prontuário como ação ao cuidado de pessoas acamadas	100,0_b_	71,7_c_	22,6_a_	0,001
	Atenção à família em caso de óbito como ação ao cuidado de pessoas acamadas	95,0_b_	24,5_a_	12,9_a_	0,001
	Discussão de casos com rede de saúde como ação ao cuidado de pessoas acamadas	85,0_b_	39,6_c_	9,7_a_	0,001
d3	Veículo para uso do serviço	75,0_b_	39,6_c_	19,4_a_	0,001
	Realização de eletrocardiograma no serviço	80,0_b_	49,1_a_	38,7_a_	0,012
	Realização de teste rápido de HIV no serviço	75,0_b_	49,1_c_	22,6_a_	0,001
	Saúde do idoso como tema de formação continuada para a equipe do serviço	55,0_b_	36,8_b_	6,5_a_	0,001
	Grupos ligados à igreja como apoio ou desenvolvimento de ações conjuntas para a pessoa idosa	30,0_b_	10,4_a_	6,5_a_	0,027
	CRAS como apoio para encaminhamentos e/ou desenvolvimento de ações conjuntas para a pessoa idosa	90,0_b_	72,6_a,b_	58,1_a_	0,045
	CREAS como apoio para encaminhamentos e/ou desenvolvimento de ações conjuntas para a pessoa idosa	55,0_b_	31,1_a_	22,6_a_	0,047

Notas: Letras diferentes representam valores estatisticamente distintos. p-valor definido pelo teste do qui-quadrado.

No domínio 2 são observadas as menores diferenças percentuais de realização entre os grupos de qualidade. A concentração de percentuais de resposta mais baixos situa-se entre os indicadores do domínio 3, focados nos equipamentos com os quais os serviços podem compartilhar ações e cuidados dirigidos aos idosos e promover formação continuada da equipe sobre a saúde do idoso – por exemplo, o Centro de Referência Especializado de Assistência Social (CREAS).

Na Tabela 3 destacam-se os indicadores que não apresentaram significância segundo o teste do qui-quadrado. Enquadram-se nessa situação 27% (42) dos 155 indicadores avaliados. Chama a atenção que a maioria deles concentra-se no domínio 3, no qual a não diferenciação entre os grupos se deve à disponibilidade de infraestrutura e, por outro lado, à falta de apoio da rede de serviços para atenção ao idoso na maior parte dos serviços.

**Tabela 3:** Distribuição percentual (%) das frequências dos indicadores de desempenho na atenção à saúde do idoso e ao envelhecimento em serviços de atenção primária, segundo domínio (d1, d2 e d3) e grupos de desempenho (G1, G2 e G3), com p-valores não significativos, 2014 (n = 157).

Domínio	Indicadores	G1	G2	G3	p
d1	Solicitação de mamografia a cada dois anos para mulheres com idade entre 50 e 74 anos, sem fatores de risco ou alteração do exame físico	50,0_a_	32,1_a_	32,3_a_	0,290
	Uso de protocolo como estratégia para detecção de violência contra idosos	20,0_b_	13,2_a,b_	3,2_a_	0,166
	Uso do Disque 100 como procedimento realizado após a identificação de violência contra idosos	20,0_a_	8,5_a_	6,5_a_	0,222
	Denúncia à autoridade policial como procedimento realizado após a identificação de violência contra idosos	45,0_a_	24,5_a_	25,8_a_	0,166
	Encaminhamento para o núcleo de apoio à saúde da família ou equipe de apoio como procedimento realizado após a identificação de violência contra idosos	25,0_b_	17,0_a,b_	3,2_a_	0,075
d2	Controle de pressão arterial e/ou glicemia em horários e dias específicos e conforme a necessidade como ação de rotina para doenças crônicas não transmissíveis (DCNT)	100,0_a_	90,6_a_	83,9_a_	0,160
	Encaminhamento para serviço de referência como estratégia aos não aderentes com DCNT	25,0_a_	26,4_a_	12,9_a_	0,293
	Realização de alta administrativa para os não aderentes com DCNT	10,0_a_	6,6_a_	6,5_a_	0,854
	Convocação de faltosos em atividades e consultas agendadas para resultados de exames alterados	73,7_a_	72,4_a_	67,7_a_	0,861
	Não utilização de protocolo no atendimento para pessoas com hipertensão arterial	30,0_a_	38,7_a_	38,7_a_	0,755
	Urina I como exame de rotina para pessoas com hipertensão arterial	100,0_a_	93,4_a_	83,9_a_	0,083
	Creatinina sérica como exame de rotina para pessoas com hipertensão arterial	100,0_a_	90,6_a_	83,9_a_	0,160
	Glicemia de jejum como exame de rotina para pessoas com hipertensão arterial	95,0_a_	95,3_a_	93,5_a_	0,928
	Colesterol total e frações (LDL e HDL) como exame de rotina para pessoas com hipertensão arterial	100,0_a_	98,1_a_	96,8_a_	0,713
	Eletrocardiograma como exame de rotina para pessoas com hipertensão arterial	100,0_a_	94,3_a_	83,9_a_	0,056
	Dispensação de manitol na atenção às pessoas com hipertensão arterial	15,0_b_	6,6_a,b_	0,0_a_	0,099
	Dispensação de succinato de metoprolol na atenção às pessoas com hipertensão arterial	20,0_a_	23,6_a_	22,6_a_	0,939
	Dispensação de cloridrato de verapamil na atenção às pessoas com hipertensão arterial	30,0_a_	19,8_a_	22,6_a_	0,592
	Dispensação de nitroprusseto de sódio na atenção às pessoas com hipertensão arterial	10,0_a_	6,6_a_	9,7_a_	0,778
	Não utilização de protocolo no atendimento para pessoas com diabetes mellitus tipo II	25,0_a_	37,7_a_	38,7_a_	0,528
	Exame anual de colesterol total e frações e triglicerídeos na atenção às pessoas com diabetes mellitus tipo II	90,0_b_	69,8_a,b_	58,1_a_	0,052
	Urina I como exame de rotina para pessoas com diabetes mellitus tipo II	100,0_b_	91,5_a,b_	80,6_a_	0,058
	Microalbuminúria como exame de rotina para pessoas com diabetes mellitus tipo II	60,0_b_	50,0_b_	29,0_a_	0,056
	Colesterol total e frações (LDL e HDL) como exame de rotina para pessoas com diabetes mellitus tipo II	100,0_a_	92,5_a_	83,9_a_	0,111
	Triglicerídeos como exame de rotina para pessoas com diabetes mellitus tipo II	95,0_a_	92,5_a_	80,6_a_	0,110
	Dispensação de insulina NPH na atenção às pessoas com diabetes mellitus tipo II	90,0_a_	79,2_a_	67,7_a_	0,157
d3	Acessibilidade no serviço para pessoas com deficiência (PCD) ou dificuldades de locomoção	85,0_a_	74,5_a_	61,3_a_	0,153
	Banheiro adaptado para pessoas com deficiência ou com dificuldades para locomoção	80,0_b_	53,8_a_	58,1%_a,b_	0,093
	Cadeira de rodas no serviço	100,0_a_	89,6_a_	83,9_a_	0,177
	Aplicação de vacina dupla adulto no serviço	90,0_b_	78,3_a,b_	64,5_a_	0,093
	Aplicação de vacina contra influenza no serviço	90,0_b_	79,2_a,b_	64,5_a_	0,082
	Realização de hemoglicoteste (HGT) no serviço	100,0_a_	99,1_a_	93,5_a_	0,115
	Exames clínicos laboratoriais colhidos no serviço	75,0_a_	67,0_a_	51,6_a_	0,174
	Acesso à rede municipal ou regional de serviços para atenção ao idoso	45,0_b_	29,2_a,b_	16,1_a_	0,082
	Associações de bairro como apoio ou desenvolvimento de ações conjuntas para a pessoa idosa	15,0_a_	5,7_a_	3,2_a_	0,212
	Centro de convivência do idoso como apoio para encaminhamentos e/ou desenvolvimento de ações conjuntas para a pessoa idosa	20,0_a_	26,4_a_	12,9_a_	0,274
	Centro-dia para idosos como apoio para encaminhamentos e/ou desenvolvimento de ações conjuntas para a pessoa idosa	5,0_a_	5,7_a_	6,5_a_	0,975
	Serviço especializado ao idoso como apoio para encaminhamentos e/ou desenvolvimento de ações conjuntas para a pessoa idosa	10,0_a_	15,1_a_	6,5_a_	0,413
	Organizações não governamentais como apoio para encaminhamentos e/ou desenvolvimento de ações conjuntas para a pessoa idosa	10,0_a_	7,5_a_	3,2_a_	0,606
	Outros serviços como apoio para encaminhamentos e/ou desenvolvimento de ações conjuntas para a pessoa idosa	20,0_b_	5,7_a_	12,9_a,b_	0,081

Notas: Letras diferentes representam valores estatisticamente distintos. p-valor definido pelo teste do qui-quadrado. Disque 100: serviço de denúncias e proteção contra violações de direitos humanos, disponível por 24 horas todos os dias.

A Tabela 4 traz os ajustes dos modelos de regressão linear simples (RLS) e múltipla (RLM) para os escores dos domínios para as variáveis de planejamento e avaliação em saúde que se mantiveram no modelo múltiplo final, ainda que outras possam ter sido ajustadas segundo o critério definido no método. Na análise das variáveis independentes que demonstraram relação com a diferenciação dos grupos de qualidade para o domínio 1 (d1), constata-se que a tipologia do serviço, os desdobramentos resultantes de avaliações e modificações induzidas por avaliações são diferenciais para compor os grupos com desempenho distinto.

**Tabela 4:** Ajustes dos modelos de regressão linear simples (RLS) e múltipla (RLM) para os escores dos domínios por variáveis de atividades gerais de planejamento e avaliação e tipologia do serviço, com base no Questionário de Avaliação e Monitoramento de Serviços de Atenção Básica 2014 (n = 157).

Domínio	Variável	RLS	RLM			
	
		p-valor	Coeficiente			

			Beta	95%CI		p-valor
d1 (r^2^aj. = 0,82)	Unidade básica de saúde tradicional	< 0.001	0.198	20.590	-5.591	0.001
	Elaboração de relatório dos problemas identificados para a secretaria municipal como desdobramento principal das avaliações	0.198	0.030	15.026	6.709	0.013
	O serviço não teve acesso aos resultados de avaliações como desdobramento principal das avaliações	0.227	0.006	11.430	9.812	0.029
	Organização da assistência como modificação induzida por avaliações	0.238	0.052	45.379	8.615	0.036
	Gerenciamento e organização da assistência como modificação induzida por avaliações	< 0.001	0.044	16.755	4.497	0.030
	Não participou de nenhuma avaliação anterior	0.001	0.003	-6.504	6.918	0.027
d2 (r^2^aj. = 0,96)	Unidade básica de saúde tradicional	0.001	-0.051	-26.845	-4.549	0.004
	Não utilização dos dados epidemiológicos para planejamento pelo serviço	0.017	-0.106	-14.023	1.282	0.006
	Organização da assistência como modificação induzida por avaliações	0.234	-0.024	-8.156	2.664	< 0.001
	Gerenciamento do serviço modificação induzida por avaliações	0.086	-0.030	-12.008	1.744	< 0.001
	Gerenciamento e organização da assistência como modificação induzida por avaliações	< 0.001	-0.009	-7.612	5.322	< 0.001
	Não houve modificações induzidas por avaliações no serviço	0.077	-0.018	-18.964	14.800	< 0.001
	Não participou de nenhuma avaliação anterior	< 0.001	0.319	42.771	90.010	< 0.001
d3 (r^2^aj. = 0,93)	Organização da assistência como modificação induzida por avaliações	0.247	3.627	18.534	63.012	< 0.001
	Gerenciamento do serviço modificação induzida por avaliações	0.043	2.729	8.451	52.981	0.007
	Gerenciamento e organização da assistência como modificação induzida por avaliações	< 0.001	3.769	19.155	61.471	< 0.001
	Não houve modificações induzidas por avaliações no serviço	0.039	2.891	10.467	55.824	0.004
	Não participou de nenhuma avaliação anterior	0.007	2.944	10.756	54.840	0.004

Fonte: Questionário de Avaliação e Monitoramento de Serviços de Atenção Básica 2014 e os autores.

Para o domínio 2 (d2), as variáveis independentes que refletem relação com a diferenciação dos grupos de qualidade estão relacionadas com a tipologia dos serviços, o uso dos dados epidemiológicos e com as modificações induzidas por avaliações, para formação dos grupos com melhores ou piores desempenhos. No domínio 3 (d3) as variáveis independentes que demonstram influência na formação dos distintos grupos de qualidade de desempenho para o conjunto de indicadores avaliados relacionam-se com as modificações induzidas por avaliações.

É importante destacar que o modelo de ajuste (r^2^) para cada um dos domínios demonstrou-se ótimo, fato corroborado por seus resultados: 0,82, 0,96 e 0,93, respectivamente.

## DISCUSSÃO

Os resultados apontam para um melhor desempenho dos serviços de APS nas ações relacionadas às doenças crônicas não transmissíveis, coincidindo com resultados de outros estudos^22 , 23^e refletindo a indiferenciação com que os serviços cumprem o escopo de ações na atenção à saúde do idoso e ao envelhecimento. As ações de prevenção e promoção foram incorporadas em poucos serviços e de modo incompleto, e a maioria deles não conta com rede de apoio para desenvolvimento de trabalho conjunto, observando-se a inexistência de uma atenção integral à saúde do idoso na amostra estudada.

Tal fato é corroborado por um estudo descritivo realizado previamente pelos autores com análise das frequências de resposta desses serviços de atenção primária sobre os indicadores avaliados. Esse trabalho identificou que, por exemplo, poucos contam com grupo de apoio para cuidadores de idosos (8,2%), uso de protocolo para atendimento dos casos de violência contra o idoso (12,1%), registro com risco diferenciado de pessoas com condições crônicas (31,2%) e inclusão da saúde do idoso como tema de educação continuada para a equipe de profissionais (33,1%)^11^.

O envelhecimento e a longevidade estão fortemente associados ao desenvolvimento de doenças crônicas, pois na realidade envelhecer sem nenhuma doença crônica é muito mais uma exceção do que uma regra^24^. Não se pode, entretanto, reduzir a velhice a um conjunto de adoecimentos nas ações desenvolvidas na APS.

Há um aparente consenso sobre a relevância de ações preventivas para o cuidado integral à saúde do adulto e principalmente do idoso; todavia, pode se observar que as práticas tendem a se restringir à assistência às DCNT de maior prevalência^5 , 24^. Não se trata evidentemente de minimizar a importância da qualidade da assistência às pessoas com doenças crônicas, mas de destacar o quanto os idosos são principalmente abordados a partir da inserção nesse conjunto de agravos, que não inclui automaticamente os cuidados necessários a eles e ao envelhecimento.

De fato, as ações realizadas pelo programa HiperDia, instituídas desde 2002 pelo Ministério da Saúde em prol das pessoas com hipertensão arterial sistêmica e diabetes mellitus^25^, foram implementadas porque as doenças crônicas representam uma das principais demandas para seguimento contínuo nos serviços de APS. Entretanto, é necessário investir na melhoria da qualidade dessa atenção, pois os serviços ainda não executam plenamente o recomendado, como em relação às ações de exame periódico de fundo de olho ou de cuidados com os pés para as pessoas com diabetes, que ocorrem em um pequeno número dos serviços avaliados^11^.

É possível constatar neste estudo que as diretrizes de prevenção e promoção da saúde acabam não sendo implementadas na atenção aos idosos e ao envelhecimento, o que ocorre também com outras etapas do ciclo de vida e demandas de saúde^26 , 27^. Nesse sentido, o monitoramento da capacidade funcional dos idosos torna-se um indicador estratégico para os serviços de saúde^23^, especialmente para os de atenção primária, que estão potencialmente instrumentalizados pela utilização de escalas para avaliação funcional e cognitiva, entre outras, disponibilizadas no *Caderno de Atenção Básica* nº 19^2^.

Estudos sobre atenção ao idoso por serviços de atenção primária realizados no Rio de Janeiro (RJ)^28^e em Santos (SP)^29^concluíram que há uma grande desarticulação da rede de serviços de saúde de um modo geral e, especialmente, uma ausência de rede de atenção à saúde do idoso, o que dificulta suprir as necessidades de saúde dessa população. As associações entre o desempenho dos serviços na atenção aos idosos e a realização de atividades indicadoras do planejamento e avaliação local mostram a importância do gerenciamento do serviço na determinação do desempenho técnico das atividades programáticas. Particularmente, a fraqueza na incorporação e utilização de dados resultantes de processos avaliativos ressalta a necessidade de fortalecimento e melhoria das práticas de monitoramento e avaliação como subsídios para a reprogramação das ações técnicas de atenção, como as direcionadas à saúde do idoso e ao envelhecimento.

Devem-se reconhecer os limites de um estudo transversal, realizado em determinadas regiões de saúde e com uma amostra de conveniência, não permitindo generalização dos resultados. Entretanto, ele traz elementos significativos diante da lacuna de avaliações e a ausência de instrumentos dirigidos especificamente para avaliar a atenção aos idosos e ao envelhecimento. Nesse sentido, mostrou-se viável e factível o uso de um instrumento que não tem esse objeto como foco, mas como parte do conjunto diversificado de ações sob responsabilidade dos serviços de APS.

É necessário avançar na oferta de práticas voltadas aos idosos pelos serviços de APS que busquem enfrentar as demandas de saúde dessa população, visando compreender as necessidades advindas do envelhecimento e colaborar para que os indivíduos alcancem a velhice com independência, autonomia e produtividade^29^, pois a maioria das enfermidades crônicas que acometem os idosos têm na própria idade seu principal fator de risco^24^. Um modelo contemporâneo com foco no envelhecimento, mas principalmente no idoso, precisa reunir um fluxo contínuo de ações de educação, promoção da saúde, prevenção de doenças evitáveis e postergação de agravos; para alcançar resultados positivos, é fundamental uma rede articulada, referenciada e com sistema de informação construído^24 , 30^.

## CONCLUSÕES

O desempenho da atenção integral ao idoso em serviços de atenção primária do SUS no estado de São Paulo mostrou-se incipiente. A incipiência é mais acentuada nas atividades de atenção para o envelhecimento saudável, enquanto as de tratamento de algumas doenças crônicas mostram desempenho relativamente melhor.

Foi possível a distinção de três grupos de serviços de desempenho decrescente segundo os três domínios da avaliação. O grupo intermediário, grupo 2, concentra a maioria dos serviços (aproximadamente 67%). O melhor grupo, grupo 1, é minoritário (aproximadamente 12%). O grupo de pior desempenho, grupo 3, concentra 31 serviços (20%) e mostra desempenho pior em todos os domínios, notadamente no de atenção ao envelhecimento saudável.

Os resultados indicam a necessidade de aprimoramento da atenção ao idoso em todos os domínios, ressaltando, porém, a urgência de investimento na melhoria do desempenho local e da rede de saúde na implementação efetiva das ações de atenção ao envelhecimento e idoso previstas nas diretrizes do Ministério da Saúde. Chama atenção também a precariedade de desempenho de vários serviços.

O quadro avaliativo desenvolvido foi capaz de caracterizar e diferenciar o desempenho atual da atenção ao idoso em serviços de APS e pode ser utilizado para o estabelecimento e disseminação de novos padrões normativos para a APS.
